# Characteristics and Management of Children With Suspected COVID-19 Admitted to Hospitals in India: Implications for Future Care

**DOI:** 10.7759/cureus.27230

**Published:** 2022-07-25

**Authors:** Santosh Kumar, Mainul Haque, Arvind Shetty, Sumesh Choudhary, Rohan Bhatt, Vivek Sinha, Balaji Manohar, Kona Chowdhury, Nadia Nusrat, Nasim Jahan, Amanj Kurdi, Zia Ul Mustafa, Johanna C Meyer, Israel A Sefah, Adnan Abdullah, Ammar Abdulrahman Jairoun, Brian Godman

**Affiliations:** 1 Periodontology and Implantology, Karnavati University, Gandhinagar, IND; 2 Pharmacology and Therapeutics, National Defence University of Malaysia, Kuala Lumpur, MYS; 3 Periodontology and Implantology, Dr DY Patil University, Navi Munbai, IND; 4 Periodontology and Implantology, Dr DY Patil University, Mumbai, IND; 5 Pediatric Dentistry, Karnavati School of Dentistry, Karnavati University, Gandhinagar, IND; 6 Pediatrics, Rajendra Institute of Medical Sciences, Ranchi, IND; 7 Periodontology and Implantology, Kalinga Institute of Industrial Technology, Bhubaneswar, IND; 8 Pediatrics, Gonoshasthaya Samaj Vittik Medical College, Dhaka, BGD; 9 Pediatrics, Delta Medical College and Hospital, Dhaka, BGD; 10 Pediatrics, Asgar Ali Hospital, Dhaka, BGD; 11 Therapeutics, University of Strathclyde, Glasgow, GBR; 12 Pharmacy, District Headquarter (DHQ) Hospital, Pakpattan, PAK; 13 Pharmacy, Division of Public Health Pharmacy and Management, School of Pharmacy, Sefako Makgatho Health Sciences University, Pretoria, South Africa, Pretoria, ZAF; 14 Pharmacy Practice, School of Pharmacy, University of Health and Allied Sciences, Keta-Dzelukope, GHA; 15 Medicine and Defence Health, Universiti Pertahanan Nasional Malaysia, (National Defence University of Malaysia), Kuala Lumpur, MYS; 16 Health and Safety, Dubai Municipality, Ajman University, United Arab Emirates, Dubai, ARE; 17 Medical and Bio Allied Health Sciences Research, Ajman University, Dubai, ARE; 18 Health Policy, Centre of Medical and Bio allied Health Sciences Research, Ajman University, Ajman, ARE; 19 Pharmacoepidemiology, Strathclyde Institute of Pharmacy and Biomedical Sciences, Glasgow, GBR

**Keywords:** outcomes, india, hospitals, guidelines, covid-19, children, antimicrobial stewardship programs, antibiotics

## Abstract

Introduction: There is a growing focus on researching the management of children with COVID-19 admitted to hospital, especially among developing countries with new variants alongside concerns with the overuse of antibiotics. Patient care can be improved with guidelines, but concerns with the continued imprudent prescribing of antimicrobials, including antibiotics, antivirals, and antimalarials.

Objective: Consequently, a need to document the current management of children with COVID-19 across India. Key outcome measures included the percentage of prescribed antimicrobials, adherence to current guidelines, and mortality.

Methodology: A point prevalence study using specially developed report forms among 30 hospitals in India.

Results: The majority of admitted children were aged between 11 and 18 years (70%) and boys (65.8%). Reasons for admission included respiratory distress, breathing difficulties, and prolonged fever. 75.3% were prescribed antibiotics typically empirically (68.3% overall), with most on the Watch list (76.7%). There were no differences in antibiotic prescribing whether hospitals followed guidelines or not. There was also appreciable prescribing of antimalarials (21.4% of children), antivirals (15.2%), and antiparasitic medicines (27.2%) despite limited evidence. The majority of children (92.2%) made a full recovery.

Conclusion: It was encouraging to see low hospitalization rates. However, concerns about high empiric use of antibiotics and high use of antimalarials, antivirals, and antiparasitic medicines exist. These can be addressed by instigating appropriate stewardship programs.

## Introduction

India rapidly introduced travel restrictions and other lockdown measures, including increasing the number of testing facilities to try and slow down the spread of COVID-19 and its impact on morbidity and mortality [1]. There was also a growing use of repurposed medicines, including hydroxychloroquine, lopinavir/ritonavir, remdesivir, and ivermectin, across countries, including India, for the prevention and treatment of patients with COVID-19 in the absence of effective vaccines and proven treatments [1-4].

However, there were concerns that some of the initial studies with repurposed medicines were typically small-scale, observational, or open-label and lacked comparator arms, alongside safety concerns [3-6]. Despite this, and based on studies including Chatterjee et al., who demonstrated that hydroxychloroquine (HCQ) significantly reduced the chances of healthcare workers getting COVID-19 [7], the Indian Council of Medical Research (ICMR) under the Ministry of Health and Family Welfare recommended HCQ for prophylaxis against COVID-19 [1,8]. Other recommended treatments included lopinavir and ritonavir [9]. However following the findings from recent studies, in September 2021, ICMR dropped HCQ and ivermectin from its clinical guidelines [10], with the updated clinical guidelines issued in January 2022 only recommending steroids, e.g., dexamethasone, and remdesivir, but only in specific circumstances, from the initially suggested repurposed medicines [11].

Compared with adults, children have a lower infection rate with COVID-19 across countries, including India, with typically milder symptoms [12-14]. Alongside this, an appreciable number have been asymptomatic [13,14]. Typical symptoms of children admitted to hospital with COVID-19 include diarrhea, fever, nausea, and respiratory infections, including coughs, with higher rates typically seen in boys than girls, with biological factors potentially playing a role [13,14]. Typically, approximately 6% to 10% of children with COVID-19 experience severe disease, with rates lower than seen in adults [13,15]. In view of this, the focus among health authorities for children during the early stages of the pandemic has been on the prevention of other infectious diseases, especially as there were concerns with low vaccination rates following lockdown measures and the subsequent impact on future morbidity and mortality [16].

However, there are growing concerns with successive waves of COVID-19 where younger patients are being treated in India and across countries, with increasing mortality seen among younger patients [12,13]. In addition, there are concerns that children are developing Kawasaki disease (KD) like symptoms/multisystem inflammatory syndromes [14,17,18] as well as experiencing hyponatremia, hypoalbuminemia, gastrointestinal changes, leucopenia, and respiratory changes with COVID-19 [14], which potentially increases admissions to intensive care units (ICU). This can be problematic since while a lower percentage of children with severe symptoms are admitted to ICUs among lower- and middle-income countries (LMICs), deaths among these hospitalized children tend to be higher [14,15]. This is reflected by mortality rates from COVID-19 among admitted children reaching 13.3% at Dhaka Shishu (Children’s) Hospital, Bangladesh, at the start of the pandemic; however, lower in later studies [14]. At the pandemic's beginning, high mortality rates (40%) were also seen at Dr. Cipto Mangunkusumo Hospital in Indonesia [19].

There are also increasing concerns about the overuse of antibiotics to treat patients with COVID-19, including children, despite limited evidence of bacterial or fungal co-infections [14,20]. This is exacerbated by the typically empiric administration of antibiotics across countries, especially among LMICs [14], which has the potential to increase antimicrobial resistance (AMR), increasing morbidity, mortality, and costs unless addressed [14,20,21].

Objectives of the study

This paper aims to document current prevalence rates, treatment approaches, and outcomes of children admitted to hospitals with suspected COVID-19 across India, building on the findings in our pilot study [22]. The results can be used to provide guidance to improve the management of children with COVID-19 in this and other future pandemics across India and wider. This is important given concerns with rising rates of AMR in India as well as concerns with the impact of the pandemic on lockdown and other measures appreciably impacting the teaching of healthcare professionals (HCPs) about the appropriate management of patients with infectious diseases, including those with COVID-19 [1,22,23].

## Materials and methods

Study setting and design

The study included a range of hospitals (N=30) across India admitting children with actual or suspected COVID-19. The hospitals were purposely selected based on their ability to provide the requested information as well as provide variation in their geography and size regarding the number of pediatric patients being treated.

The study was conducted based on standard point prevalence survey methodologies; however, adapted to meet the specific requirements of this study, building on a similar study in Bangladesh [14,24-26]. Data were gathered from July to November 2021. This included the rationale for admittance to the selected hospitals in the first place as well as subsequent admission to the pediatric intensive care units (PICUs) when needed.

Data Collection Tool and Analysis

The investigators used a paper-based collection tool in each hospital to collect the necessary patient-level data, similar to the pilot study undertaken in India and the comprehensive study in Bangladesh [14,22]. The rationale for the items contained in the data collection forms is documented in the study in Bangladesh [14]. This included breaking antibiotics prescribed into the World Health Organization (WHO) AWaRe (Access, Watch, and Reserve) classification [14,24,26]. PCR testing was provided by Xpert Xpress SARS CoV-2, Cepheid India Pvt. Ltd.

The principal investigator collected the patient-level data in each hospital, with data collection taking place on one specific day, similar to other point prevalence studies (PPS). The data were subsequently entered onto Microsoft Excel® (Microsoft Corp., Redmond, WA, US) for analysis. The Excel menu contained drop-down menus with options for admittance to the hospital and the PICUs to aid analysis, with the menu principally based on the study in Bangladesh [14]. The aggregated data were subsequently transferred to the principal investigators (SK [first author] and BG [last author]) for analysis. There was the ability to re-check and re-validate the data concerning the pertinent hospitals. Descriptive statistics were principally used to summarize the data with proportions. No attempt was made to compare and contrast the hospitals due to likely differences in their admittance criteria. A simple chi-square statistical analysis was used to assess whether there was a significant difference in antimicrobial prescribing rates between those hospitals where the principal investigator stated they did follow national guidance and those that did not, with a p-value <0.05 seen as significant.

Similar to the study in Bangladesh and the pilot study in India, medical records could be viewed up to 10 days previously. Ten days were chosen because of the anticipated low numbers of patients with COVID-19 admitted to the participating hospitals on any specific day, with most children generally asymptomatic or with milder symptoms than adults [14,15]. Alongside this, the study's principal objective was to gain a greater understanding of the current management of children with COVID-19 in India to guide future management rather than a robust assessment of the current prevalence rates of hospitalized children.

Patient, hospital anonymity, and ethical approval

The principal investigator maintained patient anonymity in each hospital throughout the study period, with only anonymized aggregated data forwarded to the principal investigators (SK and BG) for analysis. Additionally, in the final analysis, each hospital was assigned a specific number (1 to 30) to maintain anonymity further.

No parents or guardians were approached for consent since this retrospective study was based on data collated from patients’ medical records with no direct contact with children, parents, or guardians. This is in line with previous point prevalence studies undertaken by the co-authors across countries [14,24,26].

The study was conducted according to the guidelines of the Declaration of Helsinki. The co-authors orchestrated ethical approval for their hospitals, building on the initial approval orchestrated by the lead co-author. The ethical approval was obtained from Karnavati School of Dentistry Ethics Committee (KSDEC) A/907, Adalaj-Uvarsad Rd, Gandhinagar, Gujarat 382422, India (Reference No. SK- KSDEC/21-22/Apr/001, Date July 23, 2021).

## Results

We will first document the characteristics of the admitted children to the hospital who subsequently had confirmed COVID-19, their gender and ages, and rationale for admittance to the hospital as well as to any PICUs. COVID-19 was typically confirmed via polymerase chain reaction (PCR) testing among all participating hospitals.

We will subsequently discuss the current prescribing of antimicrobials among children with COVID-19, including antibiotics, anti-malarial (hydroxychloroquine [HCQ]), antiviral (remdesivir), and antiparasitic medicines (ivermectin), alongside adherence to current guidelines. Finally, documenting the current prescribing of additional therapies, including steroids among admitted children and their outcomes.

Patient characteristics

Out of the 1,606 children admitted to the 30 participating hospitals during the study period, only 15.1% had COVID-19. The majority of admitted children were aged between 11 and 18 years (70%) and boys (65.8%) (Table 1).

**Table 1:** Patient characteristics among the participating hospitals during the study period. NB: Column 2 includes children admitted during the study period and not on any specific day, with column 3 depicting the number with COVID-19 (confirmed via PCR testing)

Hospital	Total number of children admitted during the study period	Number of children with COVID-19 (no.)	% of admitted children with COVID-19	Number of boys (no.)	Number of girls (no.)	0 to 5 years of age (no.)	6 to 10 years of age (no.)	11 to 18 years of age(no.)
1	58	9	15.5%	6	3	0	3	6
2	34	7	20.6%	6	1	0	2	5
3	13	1	7.7%	1	0	0	1	0
4	67	10	14.9%	6	4	0	3	7
5	42	6	14.3%	4	2	0	4	2
6	63	15	23.8%	11	4	1	4	10
7	29	3	10.3%	2	1	0	0	3
8	67	13	19.4%	9	4	1	5	7
9	43	6	14.0%	5	1	0	5	1
10	98	22	22.4%	13	9	1	4	17
11	53	6	11.3%	3	3	1	3	2
12	160	20	12.5%	12	8	1	5	14
13	54	7	13.0%	3	4	0	0	7
14	77	11	14.3%	7	4	1	2	8
15	72	7	9.7%	5	2	2	2	3
16	21	3	14.3%	1	2	0	0	3
17	28	3	10.7%	3	0	0	1	2
18	34	6	17.6%	4	2	0	1	5
19	112	16	14.3%	9	7	0	6	10
20	91	14	15.4%	9	5	1	4	9
21	86	13	15.1%	11	2	0	1	12
22	24	3	12.5%	3	0	0	0	3
23	77	11	14.3%	8	3	0	2	9
24	35	6	17.1%	3	3	0	1	5
25	82	10	12.2%	6	4	2	2	6
26	21	3	14.3%	3	0	0	0	3
27	16	3	18.8%	1	2	0	1	2
28	18	4	22.2%	3	1	0	0	4
29	19	3	15.8%	1	2	0	0	3
30	12	2	16.7%	2	0	0	0	2
Summary characteristics	1606	243	15.1%	160	83	11	62	170

Admission characteristics

Most children were admitted with prolonged fever, breathing difficulties, coughing, and respiratory distress (Table 2). 22.2% of admitted children with COVID-19 were subsequently treated in the PICUs, with the majority admitted without ventilation support (72.2%). The principal reasons for admittance to PICUs included severe respiratory distress and low oxygen saturation (Table 2).

**Table 2:** Rationale for Admission of Children to Hospital with suspected COVID-19 and subsequently to PICU. NB: CHD = coronary heart disease; HRCT= High-resolution computed tomography; PICUs = pediatric intensive care unit.

Hospital	Primary reasons for hospital admission for children with diagnosed COVID-19	Total number of children admitted with COVID-19 to PICUs with the ventilator	Total number of children admitted with COVID-19 to PICUs without the ventilator	% requiring ventilation	The primary reason for admission to PICUs	Key underlying co-morbidities (if pertinent)
1	Prolonged fever; breathing difficulties, coughing	0	2	0	Severe respiratory distress/ low O2 saturation, shock.	Blood disorders
2	Prolonged fever; breathing difficulties/ respiratory distress, coughing, diarrhea	1	1	50%	Severe respiratory distress/ low O_2_ saturation	None
3	Prolonged fever	0	0	NA	NA	None
4	Prolonged fever, cough diarrhea	1	0	100%	Respiratory distress	None
5	Prolonged fever, severe coughing	0	1	0%	Severe respiratory distress/ low O2 saturation., shock	None
6	Prolonged fever; breathing difficulties/ respiratory distress, coughing, diarrhea, feeding difficulties, vomiting	1	2	33.3%	Severe respiratory distress/ low O2 saturation., extensive lung Involvement in HRCT	Obesity
7	Prolonged Fever; Breathing Difficulty/Respiratory Distress; Cough; Diarrhea	0	0	NA	NA	Obesity
8	Prolonged Fever; Breathing Difficulty/Respiratory Distress; Cough	2	2	50%	Severe Respiratory Distress/ Low O2 Saturation., Coagulation Disorders	Asthma
9	Prolonged Fever; Breathing Difficulties	0	1	0%	Severe Respiratory Distress/ Low O2 Saturation., Shock	Immunosuppression
10	Prolonged Fever; Breathing Difficulties/Respiratory Distress; Coughing	2	4	33.3%	Severe Respiratory Distress/ Low O2 Saturation	Thalassemia, Asthma
11	Prolonged Fever; Breathing Difficulty/Respiratory Distress; Coughing	0	2	0%	Severe Respiratory Distress/ Low O2 Saturation., Shock., Extensive Lung Involvement in HRCT	Malnutrition, CHD
12	Prolonged Fever; Breathing Difficulty/Respiratory Distress; Coughing	2	2	50%	Severe Respiratory Distress/ Low O2 Saturation., Shock	Allergies
13	Prolonged Fever; Cough; Feeding Difficulties/Vomiting	0	1	0%	Severe Respiratory Distress/ Low O2 Saturation	None
14	Prolonged Fever; Breathing Difficulties/Respiratory Distress; Diarrhea	1	2	33.3%	Severe Respiratory Distress/ Low O2 Saturation., Shock	Hypertension
15	Prolonged Fever; Breathing Difficulties/Respiratory Distress; Coughing	1	2	33.3%	Severe Respiratory Distress/ Low O2 Saturation	Complex cyanotic heart disease, hepatic encephalopathy
16	Prolonged Fever; Breathing Difficulties/Respiratory Distress	0	0	NA	NA	Asthma, cancer
17	Severe breathing difficulties	0	1	0%	Severe Respiratory Distress/ Low O2 Saturation., Shock., Extensive Lung Involvement in HRCT	Malnutrition
18	Prolonged Fever; Breathing Difficulties/Respiratory Distress; Coughing	1	1	50%	Severe Respiratory Distress/ Low O2 Saturation., Shock	Asthma
19	Prolonged Fever; Breathing Difficulties/Respiratory Distress; Feeding Difficulties/Vomiting	1	2	33.3%	Severe Respiratory Distress/ Low O2 Saturation., Extensive Lung Involvement in HRCT	Malnutrition, CHD
20	Prolonged Fever; Breathing Difficulties/Respiratory Distress; Cough; Diarrhea	0	3	0%	Severe Respiratory Distress/ Low O2 Saturation., Shock., Coagulation Disorders	Asthma
21	Prolonged Fever; Breathing Difficulties/Respiratory Distress; Cough; Diarrhea	0	3	0%	Severe Respiratory Distress/ Low O2 Saturation., Extensive Lung Involvement in HRCT	None
22	Prolonged fever, severe coughing, respiratory problems	0	0	0%	NA	None
23	Prolonged Fever; Breathing Difficulties/Respiratory Distress; Coughing	1	1	50%	Severe Respiratory Distress/ Low O2 Saturation	Thalassemia, malnutrition
24	Prolonged high fever	0	1	0%	Severe Respiratory Distress/ Low O2 Saturation	Low socio economic and compromised hygiene
25	Breathing Difficulties/Respiratory Distress	1	3	25%	Severe Respiratory Distress/ Low O2 Saturation., Shock	Previous chronic diseases, malnutrition
26	Prolonged Fever; Breathing Difficulties/Respiratory Distress; Cough; Diarrhea	0	0	NA	NA	Allergic spasms
27	Prolonged high fever	0	1	0%	Severe Respiratory Distress/ Low O2 Saturation	None
28	Prolonged Fever; Breathing Difficulties/Respiratory Distress; Cough; Diarrhea	0	1	0%	Severe Respiratory Distress/ Low O2 Saturation., Extensive Lung Involvement in HRCT	None
29	Respiratory distress; Diarrhea	0	0	NA	NA	None
30	Prolonged Fever; Breathing Difficulties/Respiratory Distress; Cough; Diarrhea.	0	0	NA	NA	None
	Summary of characteristics	27.8% admitted with ventilator	72.2% admitted w/o a ventilator			

Clinical management including antibiotics

The clinical management of children with COVID-19 among the 30 hospitals included appreciable prescribing of antibiotics (75.3% of children with COVID-19), empirically (68.3%) (Table 3). The principal antibiotic prescribed was azithromycin, with antibiotics from the WHO Watch list being prescribed in children in 76.7% of participating hospitals, typically for seven days (53.3% of participating hospitals) from three to 10 days. There was also appreciable prescribing of HCQ (21.4% of children with COVID-19), remdesivir (15.2% of children with COVID-19), and ivermectin (27.2% of children with COVID-19) (Table 3).

**Table 3:** Use of antimicrobials among children admitted to the participating hospitals in India with COVID-19. NB: Antibiotics could also be prescribed for underlying co-morbidities; CST = Culture and sensitivity testing.

Hospital	Number and % prescribed antibiotics	How antibiotics prescribed	Antibiotics principally prescribed and AWaRe classification	Route of administration	Average duration of prescriptions (days)	Were children clinically re-assessed	Number Rx HCQ (and %)	Number Rx remdesivir (and %)	Number Rx ivermectin (and %)
1	9 (100%)	Empirically	Azithromycin (W)	IV/ oral	7	Yes	2 (22%)	0	2 (22%)
2	4 (57%)	Empirically	Azithromycin (W)	IV/ oral	7	Yes	1 (14%)	2 (29%)	4 (57%)
3	1 (100%)	Empirically	Azithromycin (W)	Oral	7	Yes	0 (0%)	0 (0%)	1 (100%)
4	10 (100%)	Empirically	Azithromycin (W)	IV	5	Yes	1 (10%)	1 (10%)	3 (30%)
5	4 (67%)	Empirically	Azithromycin (W)	IV	7	Yes	1 (17%)	0 (0%)	0 (0%)
6	15 (100%)	Empirically	Piperacillin (W)	Oral, IV	5	Yes	9 (60%)	1 (7%)	0 (0%)
7	2 (67%)	Empirically	Azithromycin (W)	Oral	5	No	0 (0%)	0 (0%)	0 (0%)
8	10 (75%)	Based on CST	Azithromycin (W)/ Piperacillin (W)	IV/ Oral	7	Yes	2 (15%)	3 (23%)	2 (15%)
9	4 (67%)	Empirically	Azithromycin (W)	IV/ Oral	7	Yes	2 (33%)	1 (17%)	4 (67%)
10	17 (77%)	Empirically	Azithromycin (W)/ Piperacillin (W)	IV/ Oral	7	Yes	3 (14%)	5 (23%)	11 (50%)
11	6 (100%)	Empirically	Azithromycin (W)	IV	10	Yes	0 (0%)	1 (17%)	1 (17%)
12	17 (85%)	First empirically then after cultures	Amoxicillin (A)/ Azithromycin (W)	Oral, IV	7	Yes	4 (20%)	4 (20%)	4 (20%)
13	2 (29%)	Empirically	Piperacillin (W)	IV	7	Yes	2 (29%)	0 (0%)	0 (0%)
14	4 (36%)	Based on CST	Amikacin (A)	IV/ Oral	7	Yes	3(27%)	3 (27%)	4 (36%)
15	5 (71%)	Empirically	Amikacin (A)	IV	7	Yes	1 (14%)	0 (0%)	0 (0%)
16	1 (33%)	Empirically	Azithromycin (W)	Oral	5	Yes	0 (0%)	0 (0%)	0 (0%)
17	2 (67%)	Empirically	Amoxicillin, amoxicillin-clavulanic (A)	Oral	3	Yes	0 (0%)	1 (33%)	1 (33%)
18	4 (67%)	Empirically	Doxycycline/ Azithromycin (A/ W)	Oral, IV	5	Yes	3 (50%)	1 (17%)	3 (50%)
19	10 (63%)	Empirically	Azithromycin (W)	Oral, IV	5	Yes	1 (6%)	3 (19%)	2 (12.5%)
20	7 (50%)	Based on CST	Amikacin/ Azithromycin (A/W)	Oral, IV	7	Yes	3 (21%)	2 (14%)	4 (29%)
21	13 (100%)	Based on symptoms	Amikacin (A)	IV/ oral	5	Yes	3 (23%)	3 (23%)	6 (46%)
22	2 (67%)	Based on CST	Azithromycin (W)	Oral	5	Yes	0 (0%)	0 (0%)	3 (100%)
23	8 (73%)	Empirically	Meropenem (W)	IV, Oral	7	Yes	3 (27%)	2 (18%)	5 (45%)
24	6 (100%)	Empirically	Azithromycin (W)	IV, oral	7	Yes	5 (83%)	1 (17%)	3 (50%)
25	8 (80%)	Empirically	Meropenem (W)	Oral, IV	7	Yes	3 (30%)	1 (10%)	2 (20%)
26	3 (100%)	Empirically	Azithromycin (W)	Oral	5	Yes	0 (0%)	0 (0%)	0 (0%)
27	0 (0%)	Based on symptoms	Azithromycin (W)	Oral, IV	7	Yes	0 (0%)	0 (0%)	0 (0%)
28	4 (100%)	Empirically	Amoxicillin, amoxicillin-clavulanic (A)	IV, oral	5	Yes	0 (0%)	1 (25%)	1 (25%)
29	3 (100%)	Empirically	Azithromycin (W)	Oral	5	Yes	0 (0%)	0 (0%)	0 (0%)
30	2 (100%)	Empirically	Azithromycin (W)	Oral, IV	8	Yes	0 (0%)	0 (0%)	0 (0%)
Summary characteristics	183 (75.3%)	68.3% Empiric	76.7% Watch				21.4%	15.2%	27.2%

The length of hospital stays of admitted children varied from four to 15 days among the participating hospitals, with the vast majority of children recovering (92.2%).

Adherence to guidelines and other treatments prescribed

There was lower antibiotic prescribing to children with COVID-19 (73.4%, 141/192) among clinicians in the participating hospital who stated they were following hospital guidelines (based on national guidelines) compared with those who stated they were not following current guidelines (82.4%, 42/51). However, this was not statistically significant (p=0.189). Typically, admitted children were prescribed steroids, generally dexamethasone, alongside immunomodulators including vitamin C, D, and zinc.

## Discussion

We believe this is the first comprehensive study conducted in India documenting the management and outcomes of children hospitalized with COVID-19. The recovery rates (92.2%) mirror those seen in other countries [14,18], with full recovery seen among 80% of participating hospitals. This was appreciably improved compared with the high rates of mortality seen in Bangladesh and Indonesia at the start of the pandemic [14,19]. This may reflect increased knowledge of dealing with multisystem inflammatory syndromes in children as well as updated advice from respected groups, including the WHO and the British Medical Journal [5,14]. However, additional research is needed on this situation before anything can be said with certainty. Overall, only a small number of children were admitted to participating hospitals with COVID-19 from the total number of patients in the pediatric wards (15.1%), mirroring other recent publications [14,27]. Similar to other studies, more boys than girls were admitted with COVID-19 [13,14,27], with most over 11 years of age (Table 1). Respiratory diseases, including coughing as well as fever, were the most frequent reasons for children being admitted to the participating hospitals in India (Table 2), again similar to other studies across countries [14,15,27]. 

Encouragingly, anti-inflammatory medicines, especially dexamethasone, were being administered to children with COVID-19 in all participating hospitals, along with appreciable prescribing of Vitamin C and D as well as zinc, in line with recommendations [14,22]. However, there were a number of areas of concern. These included the appreciable prescribing of antimicrobials among the admitted children, including antibiotics and antimalarial, antiviral, and antiparasitic medicines. Overall, antibiotics were prescribed to 75.3% of children, mainly from the WHO Watch list (76.7% of participating hospitals) and typically empirically (68.3%). This is similar to the situation in Bangladesh (87.4% of admitted children were prescribed antibiotics), empiric prescribing again [14]. This is a concern as national guidelines in both countries advocate that antibiotics should only be prescribed if there is evidence or strong suspicion of bacterial infections. There is limited evidence of this to date [14,20,22,28]. Alongside this, India already is the largest or one of the largest consumers of antibiotics, with utilization rates and AMR rates continuing to rise [22,29]. Potential ways forward among hospitals in India include instigating antimicrobial stewardship programs (ASPs), which have not been implemented, alongside additional education input with modules surrounding the management of patients with COVID-19 [21,29]. ASPs and educational input have been successful in several low- and middle-income countries (LMICs), including India, improving antimicrobial prescribing [21,29]. Additional targets for quality improvement programs include encouraging greater use of culture and sensitivity testing as well as greater prescribing of Access as opposed to Watch antibiotics [14,21,24,26]. Alongside this, encouraging greater adherence to prescribing guidelines with no significant difference in antimicrobial prescribing rates among children in hospitals where clinicians stated they followed the guidelines versus those that did not. This is because studies have shown that increased adherence to published guidelines reduces unnecessary antimicrobial prescribing, which is important as more becomes known about COVID-19 and effective treatments [21,30].

Other identified areas of concern included high prescribing rates of HCQ, remdesivir, and ivermectin at 21.4%, 15.2%, and 27.2% of patients, respectively. This is despite increasing evidence of limited impact on patient outcomes as well as concerns with their prescribing in current recommendations in India [5,14,22] and contrasts with their very low use in Bangladesh [14]. However, only recently, guidelines in India stopped including HCQ and ivermectin [10]. In view of this, there is an urgent need to increase the teaching of evidence-based medicine approaches in universities in India and continued post-qualification to equip HCPs in the future better to fully appraise evolving evidence especially given the impact of the pandemic on the current education of HCPs [23]. We will be following up on areas of concern in future research projects.

Limitations of the study

There are a number of limitations to this study. These include the fact that the findings were based on a retrospective review of patients’ records without the ability to question HCPs on their actions, including prescribing antimicrobials, with the inherent limitations of this type of study. We were also constrained by the level of information that could be collected from participating hospitals. We also extended the period for patient data collection as our objective was to collect current data on the management of children with COVID-19 rather than undertaking an epidemiology study. Consequently, our study may have overestimated the prevalence rates seen for children with COVID-19. Despite these limitations, we believe our findings are robust given the number of hospitals that participated in the study.

## Conclusions

The low rates of the hospitalization of children with COVID-19 among the participating hospitals in India are encouraging, mirroring the recent findings in other countries, including Bangladesh. However, there was appreciable prescribing of repurposed antimicrobials, including antimalarials, antivirals, and antiparasitic medicines. This is a concern given the recent findings of the WHO Solidarity and the UK recovery studies showing no improved outcomes with these medicines and the possibility of patient harm. This reflects some of the guidance within national guidelines in India during the pandemic, which needs to be addressed going forward. The appreciable prescribing of antibiotics, often empiric, is also a concern even among hospital clinicians who stated they were following current guidelines. Again, this needs to be addressed given rising antibiotic consumption in India and problems with rising AMR.

Recommendations

There are a number of recommendations emanating from our findings. These include promoting evidence-based medicine in medical and pharmacy schools and continuing post-qualification. In addition, a greater use of culture and sensitivity testing before administering antibiotics, especially in patients with predominantly viral infections, including COVID-19. The instigation of local ASPs, including local antibiograms and monitoring adherence to current guidelines in future point prevalence and other studies, are also vital ways forward in India to improve future antibiotic use. Such activities should also impact rising AMR rates. We will be monitoring such activities in the future.

## References

[1] Haque M, Kumar S, Charan J (2020). Utilisation, availability and price changes of medicines and protection equipment for Covid-19 among selected regions in India: findings and implications. Front Pharmacol.

[2] Godman B, Haque M, Islam S (2020). Rapid assessment of price instability and paucity of medicines and protection for COVID-19 across Asia: findings and public health implications for the future. Front Public Health.

[3] Abubakar AR, Sani IH, Godman B, Kumar S, Islam S, Jahan I, Haque M (2020). Systematic review on the therapeutic options for COVID-19: clinical evidence of drug efficacy and implications. Infect Drug Resist.

[4] Charan J, Kaur RJ, Bhardwaj P, Haque M, Sharma P, Misra S, Godman B (2021). Rapid review of suspected adverse drug events due to remdesivir in the WHO database; findings and implications. Expert Rev Clin Pharmacol.

[5] (2022). BMJ COVID-19. https://bestpractice.bmj.com/topics/en-gb/3000201.

[6] Abena PM, Decloedt EH, Bottieau E (2020). Chloroquine and hydroxychloroquine for the prevention or treatment of COVID-19 in Africa: caution for inappropriate off-label use in healthcare settings. Am J Trop Med Hyg.

[7] Chatterjee P, Anand T, Singh KJ (2020). Healthcare workers & SARS-CoV-2 infection in India: a case-control investigation in the time of COVID-19. Indian J Med Res.

[8] (2022). Rolling out mass hydroxychloroquine prophylaxis for covid-19 in India’s slums risks eroding public trust. https://blogs.bmj.com/bmj/2020/05/01/rolling-out-mass-hydroxychloroquine-prophylaxis-for-covid-19-in-indias-slums-risks-public-trust/.

[9] Bhatnagar T, Murhekar MV, Soneja M, Gupta N, Giri S, Wig N, Gangakhedkar R (2020). Lopinavir/ritonavir combination therapy amongst symptomatic coronavirus disease 2019 patients in India: protocol for restricted public health emergency use. Indian J Med Res.

[10] Rao N (2022). As ICMR revises Covid guidelines, India can exit the thrall of ivermectin, HCQ. https://science.thewire.in/health/icmr-revises-covid-treatment-guidelines-removes-ivermectin-hydroxychloroquine/.

[11] (2022). Clinical guidance for management of adult COVID-19 patients. January.

[12] Kumar G, Mukherjee A, Sharma RK (2021). Clinical profile of hospitalized COVID-19 patients in first &amp; second wave of the pandemic: insights from an Indian registry based observational study. Indian J Med Res.

[13] US Department of Health and Human Services/Centers for Disease Control and Prevention (2020). Coronavirus disease 2019 in children - United States, February 12-April 2, 2020. MMWR Morb Mortal Wkly Rep.

[14] Chowdhury K, Haque M, Nusrat N (2022). Management of children admitted to hospitals across Bangladesh with suspected or confirmed COVID-19 and the implications for the future: a nationwide cross-sectional study. Antibiotics (Basel).

[15] Irfan O, Muttalib F, Tang K, Jiang L, Lassi ZS, Bhutta Z (2021). Clinical characteristics, treatment and outcomes of paediatric COVID-19: a systematic review and meta-analysis. Arch Dis Child.

[16] Lassi ZS, Naseem R, Salam RA, Siddiqui F, Das JK (2021). The impact of the COVID-19 pandemic on immunization campaigns and programs: a systematic review. Int J Environ Res Public Health.

[17] Malek A, Khadga M, Zahid MN (2022). Multisystem inflammatory syndrome of a neonate from a COVID-19-infected mother: a case report. Cureus.

[18] Ward JL, Harwood R, Smith C (2022). Risk factors for PICU admission and death among children and young people hospitalized with COVID-19 and PIMS-TS in England during the first pandemic year. Nat Med.

[19] Dewi R, Kaswandani N, Karyanti MR (2021). Mortality in children with positive SARS-CoV-2 polymerase chain reaction test: Lessons learned from a tertiary referral hospital in Indonesia. Int J Infect Dis.

[20] Langford BJ, So M, Raybardhan S (2021). Antibiotic prescribing in patients with COVID-19: rapid review and meta-analysis. Clin Microbiol Infect.

[21] Godman B, Egwuenu A, Haque M (2021). Strategies to improve antimicrobial utilization with a special focus on developing countries. Life (Basel).

[22] Kumar S, Haque M, Shetty A (2022). Current management of children with COVID-19 in hospitals in India; pilot study and findings. Adv Hum Biol.

[23] Sharma P, Chowdhury K, Kumar S (2022). A pilot study regarding the consequence of the COVID-19 pandemic on Healthcare Education in India and the implications. Adv Hum Biol.

[24] Mustafa ZU, Salman M, Yasir M (2022). Antibiotic consumption among hospitalized neonates and children in Punjab province, Pakistan. Expert Rev Anti Infect Ther.

[25] Saleem Z, Hassali MA, Godman B (2020). Point prevalence surveys of antimicrobial use: a systematic review and the implications. Expert Rev Anti Infect Ther.

[26] Skosana PP, Schellack N, Godman B, Kurdi A, Bennie M, Kruger D, Meyer JC (2021). A point prevalence survey of antimicrobial utilisation patterns and quality indices amongst hospitals in South Africa; findings and implications. Expert Rev Anti Infect Ther.

[27] Mansourian M, Ghandi Y, Habibi D, Mehrabi S (2021). COVID-19 infection in children: a systematic review and meta-analysis of clinical features and laboratory findings. Arch Pediatr.

[28] (2022). Ministry of Health & Family Welfare Government of India. Guidelines for Management of COVID-19 in Children (below 18 years). 202119182021.

[29] Haque M, Meyer JC, Godman B (2021). Potential ways to address antimicrobial resistance across India and wider exacerbated by COVID-19. J App Pharm Sci.

[30] Campbell SM, Meyer J, Godman B (2021). Why compliance to National Prescribing Guidelines is important especially across sub-Saharan Africa and suggestions for the future. Biomed Pharm Sci.

